# Advances in Materials Science for Precision Melanoma Therapy: Nanotechnology-Enhanced Drug Delivery Systems

**DOI:** 10.3390/pharmaceutics17030296

**Published:** 2025-02-24

**Authors:** Sivakumar S. Moni, Jobran M. Moshi, Sabine Matou-Nasri, Shmoukh Alotaibi, Yousef M. Hawsawi, Mohamed Eltaib Elmobark, Ahlam Mohammed S. Hakami, Mohammed A. Jeraiby, Ahmed A. Sulayli, Hassan N. Moafa

**Affiliations:** 1 College of Pharmacy, Jazan University, Jazan 45142, Saudi Arabia; mohamedeltaib.me@gmail.com; 2 Department of Medical Laboratory Technology, College of Nursing and Health Science, Jazan University, Jazan 45142, Saudi Arabia; 3 Health Research Centre, Jazan University, Jazan 45142, Saudi Arabia; 4 Blood and Cancer Research Department, King Abdullah International Medical Research Center, King Saud bin Abdulaziz University for Health Sciences, Ministry of National Guard-Health Affairs, Riyadh 11481, Saudi Arabia; matouepnasrisa@mngha.med.sa; 5 Biosciences Department, Faculty of the School for Systems Biology, George Mason University, Manassas, VA 22030, USA; 6 Research Center, King Faisal Specialist Hospital and Research Center, Jeddah 23433, Saudi Arabia; shmoukh.97@gmail.com (S.A.); hyousef@kfshrc.edu.sa (Y.M.H.); 7 Department of Biochemistry and Molecular Medicine, College of Medicine, Al-Faisal University, Riyadh 11533, Saudi Arabia; 8 Department Obstetrics and Gynecology, Jazan University, Jazan 45142, Saudi Arabia; ahlamhakami@jazanu.edu.sa; 9 Department of Basic Medical Science, Faculty of Medicine, Jazan University, Jazan 45142, Saudi Arabia; mmojer@jazanu.edu.sa; 10 Laboratory Department, Prince Mohammed bin Nasser Hospital, Jazan Health Cluster, Jazan 82734, Saudi Arabia; asulayli@moh.gov.sa; 11 Department of Public Health, College of Nursing and Health Sciences, Jazan University, Jazan 45142, Saudi Arabia; moafa@jazanu.edu.sa; 12 Department of Quality and Patients Safety, Jazan University Hospital, Jazan University, Jazan 45142, Saudi Arabia

**Keywords:** melanoma therapy, nanotechnology, drug delivery systems, materials science, tumor microenvironment, targeted delivery system, personalized medicine

## Abstract

Melanoma, a highly aggressive form of skin cancer, poses a major therapeutic challenge due to its metastatic potential, resistance to conventional therapies, and the complexity of the tumor microenvironment (TME). Materials science and nanotechnology advances have led to using nanocarriers such as liposomes, dendrimers, polymeric nanoparticles, and metallic nanoparticles as transformative solutions for precision melanoma therapy. This review summarizes findings from Web of Science, PubMed, EMBASE, Scopus, and Google Scholar and highlights the role of nanotechnology in overcoming melanoma treatment barriers. Nanoparticles facilitate passive and active targeting through mechanisms such as the enhanced permeability and retention (EPR) effect and functionalization with tumor-specific ligands, thereby improving the accuracy of drug delivery and reducing systemic toxicity. Stimuli-responsive systems and multi-stage targeting further improve therapeutic precision and overcome challenges such as poor tumor penetration and drug resistance. Emerging therapeutic platforms combine diagnostic imaging with therapeutic delivery, paving the way for personalized medicine. However, there are still issues with scalability, biocompatibility, and regulatory compliance. This comprehensive review highlights the potential of integrating nanotechnology with advances in genetics and proteomics, scalable, and patient-specific therapies. These interdisciplinary innovations promise to redefine the treatment of melanoma and provide safer, more effective, and more accessible treatments. Continued research is essential to bridge the gap between evidence-based scientific advances and clinical applications.

## 1. Introduction

Melanoma, the most aggressive form of skin cancer, occurs when the pigment-forming melanocytes in the basal layer of the epidermis multiply rapidly and uncontrollably due to DNA mutations triggered by ultraviolet (UV) radiation. These malignant growths often resemble moles and typically appear black or dark brown in color. The development of melanoma is triggered by a combination of genetic mutations, excessive UV radiation, and the dysregulation of the immune system. Mutations in key genes such as BRAF, NRAS, and CDKN2A promote uncontrolled cell division, while environmental factors such as chronic sun exposure and tanning bed use further increase the risk. People with fair skin, a history of frequent sunburns, or a weakened immune system are particularly at risk. If melanoma is not detected early, it can quickly metastasize to vital organs, making treatment considerably more difficult and reducing the chances of survival. In the past, melanoma posed a significant challenge to oncology, especially in advanced stages. However, with the advent of immune checkpoint inhibitors (ICI) and targeted therapies, the treatment landscape has changed dramatically over the last decade [1,2,3,4].

Melanoma therapy encompasses several categories of drugs, including targeted therapies, immunotherapies, chemotherapy, and combination treatments, each with different mechanisms and limitations. Targeted therapies focus on genetic mutations such as BRAF and MEK. BRAF inhibitors such as vemurafenib, encorafenib, and dabrafenib block the mutated BRAF V600E protein. Still, resistance develops within 6–12 months, leading to tumor recurrence and side effects such as squamous cell carcinoma of the skin, joint pain, and photosensitivity [5,6,7]. MEK inhibitors such as trametinib and cobimetinib are downstream of BRAF by targeting MEK1 and MEK2, critical components of the MAPK signaling pathway involved in melanoma progression. While these inhibitors are effective in slowing tumor growth and are often used in combination with BRAF inhibitors to delay resistance, they too eventually encounter resistance mechanisms, limiting their long-term efficacy. In addition, their use is associated with significant side effects, including diarrhea, retinal toxicity leading to visual impairment, and cardiovascular complications such as heart failure and decreased ejection fraction, making careful monitoring during treatment essential [8,9,10].

Immunotherapy, another important treatment approach, includes checkpoint inhibitors such as Programmed death-1(PD-1) inhibitors such as pembrolizumab and nivolumab, which enhance the T-cell response but can cause severe autoimmune side effects such as colitis, thyroid dysfunction, and pneumonitis. CTLA-4 inhibitors, such as ipilimumab further enhance T-cell activation but are associated with high toxicity, including hepatitis and colitis [11,12]. Cytokine therapies, such as high-dose interleukin-2 (IL-2), stimulate the immune response but have severe toxicity, including capillary leak syndrome and multi-organ failure, which limits their use [13].

Conventional chemotherapy, including dacarbazine and temozolomide, is less effective and has severe side effects, including nausea, fatigue, and bone marrow suppression, making it a less preferred option. Combination therapies are being used to improve outcomes, such as BRAF + MEK inhibitors (e.g., dabrafenib + trametinib), which delay resistance but cause fever, cardiac toxicity, and skin problems, and checkpoint inhibitor combinations (e.g., nivolumab + ipilimumab), which improve survival but significantly increase immune-related side effects. Despite these advances, drug resistance, immune-related toxicities, and limited efficacy in certain genetic subtypes remain major challenges that require further research into personalized medicine, novel drug combinations, and new therapies such as tumor vaccines and oncolytic viruses to optimize melanoma treatment [14].

Melanoma presents several therapeutic challenges due to its aggressive nature, high metastatic potential, and resistance to conventional therapies such as chemotherapy and radiation. The heterogeneity of melanoma cells and the presence of the blood-brain barrier (in the case of brain metastases) make drug administration even more difficult [15,16,17]. Materials science is addressing these challenges by developing advanced delivery systems that precisely target melanoma cells, bypassing physiological barriers, and counteract drug resistance mechanisms [18]. A key breakthrough in this field is the development of nanocarriers capable of delivering chemotherapeutics, immunomodulators, and genetic material directly to malignant cells. These engineered systems improve the solubility, stability, and bioavailability of therapeutic agents while reducing unintended effects [18]. In addition, functionalized nanoparticles tagged with ligands, antibodies, or peptides selectively recognize melanoma-specific markers such as glycoprotein 100 (gp100) and melanoma cell adhesion molecule (MCAM), thereby promoting drug accumulation in tumor tissue while minimizing systemic toxicity. Through active targeting, these specialized materials bind to overexpressed receptors such as integrins or folic acid receptors, ensuring precise drug delivery to melanoma cells.

In addition, stimuli-responsive materials that react to changes in pH, temperature, or enzymatic activity enable site-specific drug release, further improving treatments’ selectivity and therapeutic efficacy [19,20]. The resistance of melanoma to standard therapies due to genetic mutations, efflux pump activity, and adaptations of the tumor microenvironment poses major challenges for treatment. Novel materials address these problems by circumventing the resistance mechanisms through innovative approaches. The encapsulation of multiple agents in nanocarriers enables the simultaneous delivery of therapeutics, such as BRAF and MEK protein kinase inhibitors, that effectively target multiple oncogenic signaling pathways and reduce the likelihood of resistance. In addition, materials such as oxygen-generating nanoparticles modulate the tumor microenvironment by reducing hypoxia, thereby increasing the efficacy of chemotherapeutic agents and overcoming one of the major obstacles to successful melanoma treatment [21]. The future of melanoma therapy lies in the advancement of nanoparticle technology, utilizing their versatility to address challenges such as drug resistance, systemic toxicity, and treatment precision. Current nanoparticle-based approaches focus on improving drug solubility, enhancing bioavailability, and targeting therapeutic agents to melanoma cells through functionalization with ligands and peptides. These systems have been shown to be effective in reducing systemic toxicity while improving treatment outcomes. Nanoparticles are increasingly developed with enhanced capabilities for targeted and controlled drug delivery, leveraging functionalization to interact with melanoma-specific markers and integrating stimulatory mechanisms for precise drug release [22,23]. Beyond their use as carriers, the next generation of nanoparticles aims to reshape the tumor microenvironment, combat hypoxia, and optimize immune responses. Recent developments include strategies to encapsulate multiple drugs to synergize treatment pathways and overcome resistance more effectively. In addition, nanoparticles are expanding their role in personalized medicine with the prospect of combining therapeutic delivery [24,25]. Future advances in nanoparticle technology aim to integrate multifunctional capabilities, including stimuli-responsive delivery mechanisms and theragnostic applications that combine therapy and real-time imaging. Incorporating nanoparticles into immunotherapy and personalized medicine will transform melanoma treatment by enabling tailored interventions that consider individual patient characteristics and tumor behavior, thereby improving the efficacy and safety of melanoma therapies.

## 2. Therapeutic Targeting

Nanotechnology, the manipulation of materials at the nanoscale, has revolutionized numerous scientific disciplines, particularly pharmaceuticals. In cancer therapy, nanotechnology has enabled the development of advanced drug delivery methods that increase therapeutic efficacy and reduce adverse effects. Nanoparticles, nanocarriers, and nano-formulations are crucial in addressing the shortcomings of conventional cancer therapies, such as limited specificity, resistance to multiple drugs, and systemic toxicity [26,27]. The main goal of nanotechnology in cancer therapy is to ensure that therapeutic agents reach the tumor efficiently while sparing healthy tissue [28,29]. Nanotechnology is revolutionizing cancer treatment by overcoming the limitations of conventional therapies and advancing precision medicine. Nanoscale materials such as nanoparticles and nanocarriers enable the precise delivery of therapies to cancer cells, reducing systemic toxicity and adverse effects on healthy tissue. These technologies exploit unique features of the tumor microenvironment, such as abnormal vasculature, hypoxia, and acidic pH levels, to enhance drug accumulation at the tumor site. With advanced properties such as controlled release, active targeting, and responsiveness to stimuli such as pH changes, enzymes, or magnetic fields, nanoparticles maximize therapeutic efficacy. In addition, nanotechnology facilitates theranostics, i.e., the combination of therapy and diagnostic imaging for real-time tracking of drug distribution and treatment success. By overcoming biological barriers, adapting to tumor heterogeneity, and integrating personalized medicine, nanotechnology offers transformative solutions that improve efficacy, reduce side effects, and support safer, patient-specific cancer therapies [30,31].

### 2.1. Passive Targeting

**Figure 1:**
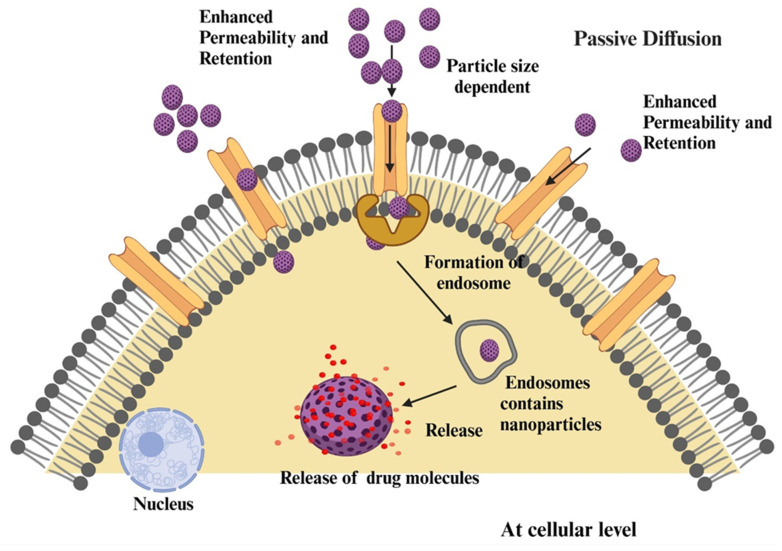
Passive targeting of nanoparticles at the cellular level. This figure was created with BioRender.com, Bio Render, Canada.

Passive targeting in cancer nanotechnology utilizes the enhanced permeability and retention (EPR) effect, a phenomenon due to structural and functional abnormalities of tumor vasculature, such as leaky blood vessels and impaired lymphatic drainage, allowing nanoparticles to selectively accumulate in the tumor microenvironment (Figure 1). This approach facilitates targeted drug delivery to cancer tissue, but its efficiency is often hampered by tumor heterogeneity, dense extracellular matrix, and increased interstitial fluid pressure, which limits drug penetration and distribution. Optimization strategies have been developed to overcome these challenges, including customization of nanoparticle design through size, shape, and surface modifications such as PEGylation, the use of controlled release mechanisms such as pH-sensitive or enzyme-responsive systems to normalize tumor vasculature, and improve EPR efficiency. Despite these challenges, passive targeting remains a fundamental strategy in cancer therapy, enabling nanotechnology delivery systems to exploit tumor-specific properties for improved therapeutic outcomes [32,33,34,35].

Tumors stimulate rapid angiogenesis to meet their need for oxygen and nutrients. However, the resulting blood vessels are disorganized, have an irregular shape, have large endothelial gaps, and have a poorly developed basement membrane. These characteristics make the tumor vessels very permeable, allowing nanoparticles and macromolecules to escape from the bloodstream and accumulate in the tumor interstitium. At the same time, tumors lack efficient lymphatic drainage, which prevents the removal of these therapeutic agents and leads to their prolonged retention in the tumor. Taken together, these features create a microenvironment in which drugs and nanoparticles preferentially concentrate in tumor tissue compared to normal tissue. The EPR effect improves drug efficacy by enabling higher local drug concentrations while minimizing systemic side effects. However, the efficacy of the EPR effect varies by tumor type and patient and is influenced by factors such as vascular density, stromal composition, and interstitial fluid pressure. Poorly vascularized tumor regions and high interstitial fluid pressure can limit drug penetration, resulting in uneven distribution of therapeutic agents. To overcome these challenges, researchers are optimizing the design of nanoparticles and exploring vaso-modulating agents to improve drug delivery efficiency. Combining the EPR effect with active targeting strategies, in which ligands on nanoparticles are bound to specific tumor markers, further increases precision. Despite its limitations, the EPR effect remains a cornerstone of nanomedicine and targeted cancer therapies, offering a way to improve the efficacy and safety of cancer treatments [36,37,38]. Tumor angiogenesis triggered by accelerated growth leads to disorganized, highly permeable blood vessels that facilitate extravasation and accumulation of nanoparticles (10–200 nm) in the tumor interstitial spaces [39,40,41,42]. Impaired lymphatic drainage increases retention and prolongs drug concentration at the tumor. Successful utilization of the EPR effect requires careful nanoparticle design. The ideal size prevents premature elimination by renal filtration or absorption by the mononuclear phagocyte system [43,44]. Shape optimization, including spherical or rod-shaped nanoparticles, affects biodistribution and tumor infiltration, while surface modifications such as PEGylation improve circulation time and evade immune recognition [45,46]. Controlled release mechanisms, such as pH-sensitive or enzyme-responsive systems, enhance targeted efficacy. Passive targeting offers advantages such as selective drug accumulation, reduced systemic toxicity, broad applicability to various solid tumors, and simpler development processes than active targeting based on ligand receptors [28,47]. Nevertheless, limitations such as variability of the EPR effect, obstacles such as dense extracellular matrix, increased interstitial pressure, and reduced efficacy in non-solid tumors pose significant problems. Advanced techniques such as tumor priming, passive and active targeting integration, and stimuli-responsive nanoparticles are currently being investigated to overcome these challenges [48,49]. Although passive targeting is essential for the delivery of nanoparticles to tumors, ongoing advances in nanoparticle development and the incorporation of tumor biology are critical to overcoming these difficulties and expanding their application in cancer treatment.

### 2.2. Active Targeting

**Figure 2:**
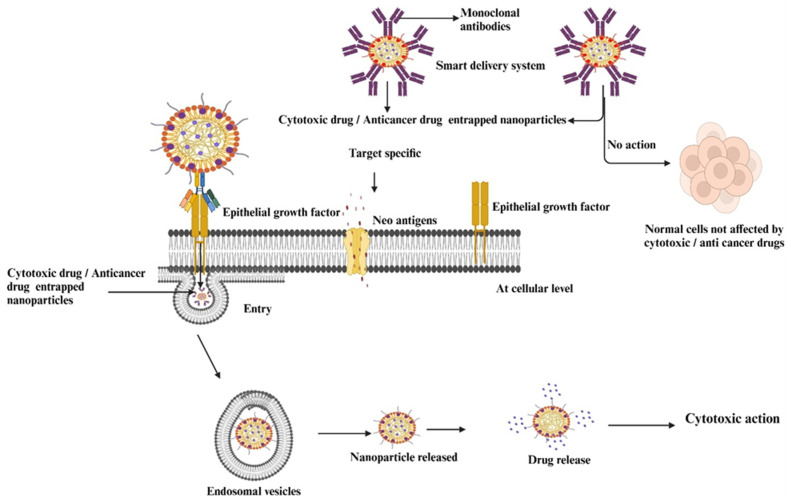
Active targeting of nanoparticles at the cellular level. This figure was created with BioRender.com, Bio Render, Canada.

The active targeting of nanoparticles in cancer therapy represents a significant breakthrough in modern oncology. The focus is on the precise delivery of therapeutics directly to cancer cells while sparing healthy tissue from unintended side effects (Figure 2). This innovative approach is based on the functionalization of nanoparticles with specific ligands, including antibodies, peptides, aptamers, or small molecules that have a high affinity for receptors that are overexpressed on the surface of cancer cells [50,51,52]. By exploiting the unique molecular signatures of tumors, e.g., the overexpression of specific receptors such as folate, transferring, or epidermal growth factor receptors, nanoparticles can target cancer cells with remarkable precision. This targeted delivery system increases the therapeutic efficacy of cancer drugs and significantly reduces off-target effects, which are a significant limitation of conventional chemotherapy [53,54]. The process of active targeting begins with the development of nanoparticles that contain functional components capable of recognizing and binding to cancer-specific biomarkers. These biomarkers are proteins, glycoproteins, or other molecular entities that are uniquely or abundantly present on cancer cells. For example, nanoparticles conjugated with folic acid target the folic acid receptors that are often overexpressed in ovarian, breast, and lung cancers. Similarly, antibodies such as trastuzumab can be conjugated with nanoparticles to attack HER2-positive breast cancer cells. Binding these ligands to the appropriate receptors ensures that the therapeutic payload is selectively delivered to the tumor, increasing the accumulation of the drug in cancer tissue while reducing systemic toxicity [55,56].

Active targeting not only improves drug delivery but also therapeutic outcomes by enabling the use of advanced therapeutic modalities such as gene therapy, photothermal therapy, and immunotherapy. Nanoparticles can be loaded with multiple therapeutic agents or combined with imaging agents to create multifunctional platforms capable of simultaneous therapy and diagnostic imaging—a concept known as theragnostics. This approach facilitates real-time monitoring of drug delivery and treatment response and provides valuable insights into therapy efficacy [57,58]. The development of nanoparticles that respond to stimuli adds another level of specificity to active targeting. These nanoparticles are designed to release their active agents in response to the microenvironment of tumors, such as acidic pH, hypoxia, or the presence of certain enzymes. For example, nanoparticles that respond to the acidic pH of the microenvironment tumor can undergo structural changes or degradation, resulting in a controlled release of the encapsulated drug. This ensures that the therapeutic effect remains limited to the tumor and the effects on normal tissue are reduced to a minimum [59,60]. Despite their promising potential, the clinical implementation of active targeting strategies faces several challenges. Ensuring the biocompatibility and stability of nanoparticles in the biological environment is crucial for their success. In addition, issues such as immune response, off-target interactions, and tumor heterogeneity can complicate the application of this technology. Overcoming biological barriers, such as the EPR effect, remains a significant hurdle, as not all tumors have the same degree of vascular permeability. Researchers are actively exploring strategies to optimize the pharmacokinetics and biodistribution of nanoparticles to maximize their therapeutic potential [61,62]. Recent advances in nanotechnology have driven the development of novel materials and designs for active targeting. For example, integrating magnetic or ultrasound-responsive elements into nanoparticles has enabled the remote control of drug release. Researchers are also exploring personalized medicine approaches to tailor nanoparticle formulations to individual patient profiles based on their tumors’ genetic and molecular characteristics. This customization aims to improve therapeutic outcomes by tailoring treatment strategies to the unique molecular traits characterizing each patient’s cancer [63,64].

### 2.3. Stimuli-Responsive Targeting

Stimuli-responsive nanoparticles represent a significant advance in precision cancer therapy, as they respond to specific internal or external triggers and ensure the release of drugs predominantly into the tumor microenvironment [65,66]. Internal stimuli-responsive systems take advantage of the unique biochemical properties of tumors to achieve targeted drug activation. For example, pH-responsive nanoparticles such as polymeric micelles and mesoporous silica nanoparticles exploit the acidic environment of tumors to release their active ingredients selectively [67]. Similarly, redox-responsive nanoparticles utilize the increased glutathione (GSH) level in cancer cells to achieve intracellular drug release. Enzyme-responsive systems target overexpressed tumor-specific enzymes such as matrix metalloproteinases (MMPs), enabling precise release at the tumor site [68]. External stimuli-responsive nanoparticles utilize externally applied triggers to achieve controlled drug release, enabling spatial and temporal precision of therapy. Light-activated nanoparticles are used in photodynamic and photothermal therapies, where specific wavelengths of light induce drug release or generate heat for tumor ablation [69]. Magnetically reacting nanoparticles are directed to the tumor using external magnetic fields, enabling targeted therapy with minimal systemic exposure [70]. Ultrasound-triggered nanoparticles use focused ultrasound to generate localized heat or mechanical disruption to ensure the precise release of therapeutic agents at the tumor site. These stimuli-responsive systems overcome the limitations of conventional therapies by improving selectivity, reducing systemic toxicity, and increasing therapeutic efficacy [71]. Their dual ability to respond to internal and external triggers enables customizable treatment approaches tailored to the tumor’s specific characteristics and the patient’s needs [72]. However, challenges such as optimizing sensitivity to stimuli, ensuring stability during circulation, and scaling up production remain critical areas for ongoing research and development in this field.

### 2.4. Multi-Stage Targeting

Multi-stage targeting represents a breakthrough innovation in nanotechnology that specifically addresses the challenges of effective drug delivery in melanoma therapy. Due to the highly aggressive nature of melanoma and the heterogeneous tumor microenvironment characterized by dense extracellular matrices and poorly vascularized regions, conventional nanoparticle-based treatments are not suitable to achieve sufficient drug penetration. Multi-stage targeting offers a dynamic solution using nanoparticles that transform sequentially to overcome these obstacles. Initially, larger nanoparticles are used to maximize circulation time and accumulate in the tumor through the EPR effect, taking advantage of the tumor’s leaky vasculature. Once in the tumor microenvironment, these particles break down into smaller fragments through stimulatory mechanisms such as pH sensitivity, enzymatic activation, or external triggers such as heat or light. This fragmentation facilitates deeper penetration into the interstitial spaces of the tumor and enables the delivery of therapeutics to regions that are otherwise inaccessible, including poorly perfused and hypoxic areas. By precisely localizing and evenly distributing agents, this approach not only increases therapeutic efficacy against melanoma but also significantly reduces off-target effects and systemic toxicity. In addition, the ability of the multistage nanoparticles to dynamically adapt to the tumor microenvironment underscores their potential as a transformative strategy in precision medicine that addresses the unmet clinical need of patients with advanced melanoma [73,74]. Once localized in the tumor, these particles transform into smaller fragments that allow deeper penetration into the interstitial tumor and even reach poorly vascularized areas. This dynamic size change ensures a comprehensive distribution of the drug in the tumor, significantly improving therapeutic efficacy and minimizing systemic toxicity [74]. Applications of multistage targeting have demonstrated its potential in various therapeutic scenarios. For example, protease-activated nanoparticles shrink in response to proteases overexpressed in the tumor microenvironment, enhancing their ability to move in the dense extracellular matrix. Similarly, resizable nanoparticles are designed to switch between different sizes at certain stages of drug delivery, balancing the need for initial tumor accumulation and subsequent deep penetration. These systems not only improve the distribution of chemotherapeutic agents but also the efficacy of combination therapies, such as the simultaneous administration of drugs and immunomodulators. By overcoming the dual challenge of targeting and interstitial penetration, multi-stage targeting represents a transformative step in the advancement of nanoparticle-based cancer therapies and paves the way for more effective and personalized therapeutic strategies [75].

## 3. Nanotechnology Platforms

Nanoparticle technology represents a breakthrough approach to melanoma therapy as it enables targeted, controlled, and highly efficient delivery of therapeutics. Its innovative platforms significantly improve the solubility, stability, and targeting of drugs, revolutionizing the treatment landscape. Recent advances in nanoparticle technology for melanoma therapy have focused on the development and application of nanoparticle formulations such as liposomes, dendrimers, micelles, and polymeric nanoparticles, all of which uniquely contribute to the optimization of therapeutic outcomes.

### 3.1. Liposomes

Liposomes are lipid-based spherical vesicles that have transformed the delivery of drugs in cancer therapy, including melanoma. Their unique structure, consisting of one or more phospholipid bilayers encapsulating an aqueous core, enables the uptake of both hydrophilic and hydrophobic drugs. This dual capability increases the versatility of liposomes and enables the delivery of a broad spectrum of therapeutic agents [76]. Liposomes are highly biocompatible due to their phospholipid composition, which mimics biological membranes, making them safe for systemic administration with minimal toxicity. They are biodegradable and degrade naturally through enzymatic hydrolysis and interaction with biological fluids, ensuring safe excretion [77]. Their immunogenicity depends on their composition, surface charge, and size, with modifications such as PEGylation reducing immune recognition and prolonging circulation time [78,79]. Liposomes efficiently load drugs by encapsulating hydrophilic molecules in their aqueous core and hydrophobic drugs in their lipid bilayer, enabling versatile drug delivery. Their permeability is adjustable, with modifications such as cholesterol incorporation increasing membrane stability and reducing premature drug leakage. They significantly improve therapeutic efficacy by increasing solubility, stability, and targeted drug delivery, reducing systemic toxicity while ensuring high drug accumulation at disease sites. Liposomal formulations also improve treatment adherence by reducing dosing frequency and minimizing side effects. In addition, they improve bioavailability by protecting drugs from enzymatic degradation, promoting cellular uptake, and facilitating controlled release, making them highly effective for poorly soluble and rapidly metabolized drugs [79]. These properties make liposomes a promising platform for drug delivery, including cancer therapy, vaccine delivery, and antimicrobial treatments.

Melanoma, an aggressive and often therapy-resistant form of skin cancer, requires advanced treatment strategies that target tumor cells while minimizing systemic toxicity. Liposomes are particularly suitable in this regard as their composition can be modified to achieve site-specific delivery. Functionalizing their surface with polyethylene glycol (PEG) improves circulation time, while conjugation with targeted ligands such as antibodies or peptides increases specificity for melanoma cells. The effect of EPR facilitates the accumulation of liposomes in tumor tissue, making them an ideal vehicle for the delivery of chemotherapeutics, immunotherapeutics, and RNA-based therapies, while minimizing off-target effects and improving therapeutic outcomes [80,81,82,83,84]. In melanoma therapy, liposomes offer solutions to key challenges such as poor penetration of drugs into the tumor, systemic toxicity of conventional treatments, and resistance to multiple drugs. Liposomal formulations such as Doxil, which contains doxorubicin, have effectively reduced cardiotoxicity while maintaining antitumor activity. Similarly, liposomes loaded with paclitaxel improve drug solubility and allow higher drug concentrations in melanoma cells, improving therapeutic outcomes [85,86,87]. In addition to traditional chemotherapeutics, liposomes offer great potential for the delivery of innovative therapies, including RNA-based molecules such as siRNA and miRNA. These molecules target genetic abnormalities that cause melanoma, such as BRAF mutations, by silencing oncogenes or restoring the functions of tumor suppressor genes [88,89,90]. Liposomes have also advanced immunotherapy by facilitating the targeted delivery of immune checkpoint inhibitors or cytokines directly into the tumor microenvironment. This approach enhances immune responses against melanoma cells while minimizing systemic immune-related side effects. In addition, the specificity of liposomal delivery systems is enhanced by the inclusion of ligands such as integrin-binding peptides or monoclonal antibodies that recognize melanoma-specific markers, such as vemurafenib, ipilimumab, and pembrolizumab. This targeted approach reduces collateral damage to healthy tissue, further optimizing the efficacy and safety [91,92,93]. The future of liposomes in melanoma therapy will be determined by advances in stimuli-responsive delivery systems and personalized medicine. Researchers are pioneering the development of pH-sensitive, thermosensitive, and enzyme-sensitive liposomes that release therapeutic agents in response to specific triggers in the tumor microenvironment. The pH-sensitive liposomes, for example, take advantage of the acidic conditions of tumors to ensure localized and precise drug release and minimize off-target effects [94,95,96,97].

### 3.2. Dendrimers

Dendrimers, highly branched nanoscale polymers, are emerging as versatile tools for melanoma therapy due to their precise architecture, high drug loading capacity, and multifunctional surface groups [98]. Dendrimers are highly biocompatible due to their well-defined, branched structure, which allows precise functionalization to minimize toxicity and improve biological interactions [99]. Their biodegradability depends on their core and surface chemistry, with certain types of dendrimers, such as polyester-based dendrimers, degrading naturally in biological environments, while others, such as poly(amidoamine) (PAMAM), may require modification to improve the rate of degradation [100,101].

Immunogenicity depends on the surface modifications; unmodified dendrimers may elicit immune responses, but PEGylation or functionalization with biocompatible components may reduce immunogenic effects. Dendrimers have a high drug loading capacity due to their internal cavities and functionalized surface groups and allow encapsulation or covalent binding of both hydrophilic and hydrophobic drugs. Their permeability is influenced by size, surface charge, and functionalization, with cationic dendrimers showing improved cellular uptake but potential cytotoxicity, while neutral or anionic dendrimers offer better biocompatibility. They enhance the therapeutic effect by improving drug solubility, protecting against enzymatic degradation, enabling controlled release, and facilitating targeted drug delivery through surface modifications. The ability of dendrimers to prolong the circulation of the drug, reduce the frequency of administration, and minimize systemic toxicity improves treatment adherence and makes them suitable for various routes of administration, including intravenous, transdermal, and ocular delivery. Dendrimers improve bioavailability by promoting drug solubility, cellular uptake, and site-specific accumulation, especially in cancer therapy, gene delivery, and antimicrobial applications, making them a promising nanoplatform for advanced drug delivery [102,103]. These structures can be tailored with ligands, antibodies, or peptides to target melanoma-specific biomarkers, such as BRAF mutations and MC1R, enabling selective drug delivery while minimizing off-target effects.

Dendrimers improve the solubility and stability of chemotherapeutics, such as doxorubicin and paclitaxel and enable combination therapies or the delivery of siRNA to disrupt critical signaling pathways, such as MAPK and PI3K/AKT [104]. While challenges, such as cytotoxicity, production costs, and scalability remain persistent, advances in biocompatible modifications, such as PEGylation are improving their safety and efficacy, making dendrimers a promising platform for targeted and personalized melanoma treatment [105]. Dendrimers are also gaining attention for their role in immunotherapy, as they can serve as vaccine carriers or adjuvants to stimulate antigen-presenting cells and boost anti-tumor immunity. Their controlled size and precise functionalization enable efficient presentation of tumor-associated antigens, enhancing dendritic cell activation and T cell responses. In addition, their use in photodynamic and photothermal therapies is being investigated by conjugating them with photosensitizers or near-infrared dyes, enabling targeted destruction of melanoma cells with minimal damage to surrounding tissue [106,107].

### 3.3. Micelles

Micelles, self-assembled nanoscale structures formed from amphiphilic molecules, have attracted considerable attention in melanoma therapy due to their ability to encapsulate hydrophobic drugs and improve their solubility, stability, and targeted delivery [108,109]. These nanocarriers are particularly effective in overcoming the challenges of conventional therapies by improving the bioavailability and therapeutic index of chemotherapeutic agents, such as paclitaxel and doxorubicin. Micelles can be functionalized with targeted ligands, antibodies, or peptides that recognize melanoma-specific biomarkers, such as overexpressed integrins or MC1R, enabling selective delivery to tumor cells while minimizing systemic toxicity. Stimuli-responsive micelles, which release their payload in response to triggers, such as pH, temperature, or enzymes in the tumor microenvironment, increase precision and reduce off-target effects [110].

Biodegradability depends on the type of surfactant or polymer used; natural and biodegradable polymers enhance degradation and ensure safe elimination. The immunogenicity of micelles is generally low, but surface modifications, such as PEGylation, further reduce immune recognition and prolong circulation [111]. Micelles have excellent drug loading capacity, especially for hydrophobic drugs encapsulated in the core, while hydrophilic drugs can sometimes be incorporated into the shell or linked by chemical conjugation. Their permeability is influenced by size, surface charge, and composition, with small micelles efficiently penetrating biological barriers and facilitating intracellular drug delivery. Micelles improve therapeutic efficacy by increasing solubility, stability, and targeted release of drugs, resulting in increased efficacy, and reduced systemic toxicity, especially for anticancer drugs and poorly water-soluble drug formulations. They also improve compliance by enabling controlled and sustained drug release, reducing dosing frequency, and minimizing adverse effects. In addition, micelles improve bioavailability by protecting drugs from enzymatic degradation, promoting solubilization, and enabling efficient absorption at the target site [112,113]. These benefits make micelles an effective platform for drug delivery in oncology. In addition to the delivery of chemotherapeutics, micelles are increasingly used for combination therapies, e.g., for the simultaneous delivery of drugs and siRNA to inhibit signaling pathways, such as MAPK and PI3K/AKT, which are critical for melanoma progression and resistance. Micellar systems also play a role in photodynamic therapy, where they carry photosensitizers that generate reactive oxygen species upon light activation, effectively killing melanoma cells. Based on their small size and biocompatibility, they can penetrate deep into the tumor and bypass the reticuloendothelial system, ensuring prolonged circulation and efficient tumor accumulation. Despite their potential, challenges, such as stability under physiological conditions and scalability remain persistent. However, ongoing advances in micelle design, including cross-linked micelles and polymeric modifications, are addressing these limitations, and solidifying their role as a promising platform for targeted and effective melanoma therapy [113].

### 3.4. Polymeric Nanoparticles

Polymeric nanoparticles have proven to be an effective tool in melanoma therapy, offering innovative solutions to overcome the limitations of conventional treatments, such as systemic toxicity, drug resistance, and poor tumor response. These nanoparticles, typically between 10 and 200 nanometers in size, are composed of biocompatible and biodegradable polymers, such as poly(lactic-co-glycolic acid) (PLGA), polylactic acid (PLA), chitosan, and polycaprolactone (PCL). Their versatile structure makes it possible to encapsulate both hydrophilic and hydrophobic active ingredients, ensure controlled and sustained drug release and improve the therapeutic index of anti-melanoma agents [114,115,116,117,118,119].

The biodegradability of polymer nanoparticles is primarily determined by the type of polymer. Natural polymers are degraded enzymatically, whereas synthetic polymers are degraded by hydrolysis, which enables controlled drug release and safe excretion. Immunogenicity is influenced by surface properties; unmodified nanoparticles can trigger immune responses, while PEGylation or functionalization with biomolecules reduces immune recognition and thus prolongs circulation time. Polymeric nanoparticles offer a high drug loading capacity and allow the encapsulation of both hydrophilic and hydrophobic drugs in their matrix or core-shell structure. This design supports sustained and controlled release of drugs. Their permeability is determined by factors, such as size, surface charge, and polymer composition, optimizing transport across biological membranes and improving cellular uptake, especially in tumor and inflamed tissue. These nanoparticles improve therapeutic efficacy by increasing drug solubility, stabilizing unstable molecules, enabling targeted and controlled release, and minimizing off-target toxicity. In addition, they can extend the half-life of drugs, reduce the frequency of administration, and support non-invasive routes of administration, such as oral, nasal, and transdermal delivery, which improves patient compliance. Polymeric nanoparticles protect the encapsulated drug from premature degradation, prolong circulation time, and facilitate selective targeting of melanoma cells through both passive and active targeting mechanisms [118,119,120,121]. Table 1 shows the polymeric nanoparticles that have been developed for melanoma therapy.

### 3.5. Hydrogels

Hydrogels have shown promise as a platform for melanoma therapy by overcoming the challenges associated with drug delivery, bioavailability, and systemic toxicity [135,136]. These three-dimensional polymer networks have a high-water retention capacity, making them biocompatible and suitable for local, sustained drug release. Conventional melanoma treatments, including chemotherapy, targeted therapies, and immunotherapy, often suffer from rapid systemic clearance, off-target toxicity, and poor tumor penetration [136]. The biodegradability of hydrogels is influenced by their polymer composition, with natural hydrogels, such as alginate, chitosan, and hyaluronic acid, being subject to enzymatic degradation, while synthetic hydrogels, such as polyacrylamide and polyethylene glycol can be engineered for controlled and adjustable degradation [137,138].

The immunogenicity of hydrogels varies; natural polymers generally have low immunogenicity, while synthetic hydrogels may need to be modified, e.g., by PEGylation or functionalization with biocompatible molecules, to reduce immune responses [139]. Hydrogels offer excellent drug loading capacity and allow the encapsulation of hydrophilic and hydrophobic drugs, proteins, and biomolecules in their porous structure, enabling sustained and controlled drug release. Their permeability is adjustable depending on cross-linking density, polymer composition, and source properties and enables controlled diffusion of drugs and nutrients while preventing premature drug release. Hydrogels improve therapeutic efficacy by enabling localized, sustained, and responsive drug delivery, protecting bioactive molecules from degradation, and reducing systemic toxicity. Adherence to therapy is improved by their ability to provide long-term drug release, reduce the frequency of administration, and enable minimally invasive delivery, such as in transdermal patches, injectable formulations, and wound dressings. In addition, hydrogels improve bioavailability by maintaining drug stability, improving retention at target sites, and facilitating controlled absorption, making them highly effective for wound healing, tissue engineering, ophthalmic drug delivery, and localized cancer therapy [140].

The integration of nanotechnology with hydrogels has led to the development of nanoparticle-loaded hydrogel systems that improve the solubility and stability of drugs while enabling deeper tumor penetration. These hybrid platforms are particularly suitable for combination therapies where multiple therapeutic agents, such as BRAF and MEK inhibitors, checkpoint inhibitors, and cytokines, are administered together to achieve synergistic effects [141,142]. The use of injectable hydrogels has further improved melanoma treatment by enabling minimally invasive delivery and precise targeting of tumor morphology [143]. Functionalized hydrogels equipped with targeted ligands or antibodies have been designed to recognize melanoma-specific biomarkers, such as glycoprotein 100 (gp100) and melanoma cell adhesion molecule (MCAM), ensuring better accumulation of the drug in the tumor microenvironment while reducing systemic side effects.

In addition to their role in direct therapy, hydrogels have revolutionized melanoma research through the development of 3D tumor models that better replicate the tumor microenvironment than conventional 2D cultures [144,145]. These biomimetic hydrogel scaffolds facilitate drug screening, improve the predictive accuracy of preclinical studies, and accelerate the discovery of new melanoma treatments. By incorporating gene therapy approaches into hydrogels, their therapeutic potential has been further expanded. For example, siRNA, CRISPR/Cas9, and microRNA can be localized and delivered in a sustained manner to silence oncogenes, modify immune responses, or reverse mechanisms of drug resistance [146]. Hydrogels have also contributed to the improvement of cancer immunotherapy by providing a platform for the controlled release of immune checkpoint inhibitors, cytokines, and tumor-specific vaccines, thereby reducing immune-related side effects and improving patient response. The advent of stimuli-responsive hydrogels, which release therapeutic agents in response to tumor-specific signals, represents the next stage of precision medicine for melanoma treatment.

### 3.6. Metallic Nanoparticles

Metallic nanoparticles (MNPs), including gold, silver, and iron oxide, have revolutionized melanoma therapy due to their unique properties, such as a high surface-to-volume ratio and adjustable size. Gold nanoparticles (AuNPs) enable precise drug delivery and photothermal ablation by targeting melanoma-specific biomarkers, such as MC1R [144,145]. In contrast, silver nanoparticles (AgNPs) generate reactive oxygen species (ROS) for direct cytotoxicity and synergize with chemotherapeutic agents. Superparamagnetic iron oxide nanoparticles (SPIONs) improve the detection of melanoma using high-resolution MRI and support early diagnosis [146,147,148]. In addition, MNPs are being explored in immunotherapy as adjuvants or carriers to enhance T-cell responses and combat drug resistance by targeting cancer stem cells and key pathways. Despite challenges, such as biocompatibility and toxicity, MNPs hold immense potential for precise, effective, and personalized melanoma treatments.

Future research on MNPs will focus on improving biocompatibility, reducing toxicity, and improving their therapeutic and diagnostic applications. Surface modifications with biopolymers, peptides, and antibodies will be explored to increase tumor specificity, minimize off-target effects, and improve turnaround time. Gold and silver nanoparticles are being developed for combined photothermal and photodynamic therapies to maximize the destruction of melanoma cells while reducing damage to surrounding healthy tissue. In addition, SPIONs are increasingly being integrated into multifunctional platforms for simultaneous imaging and therapy, enabling real-time tracking of drug distribution and tumor response [148,149,150,151]. Researchers are also investigating MNPs in combination with immunotherapies targeting immune checkpoint signaling pathways to enhance T-cell responses and overcome melanoma resistance. Despite these advances, it remains a challenge to understand the long-term toxicity, biodistribution, and excretion pathways of MNPs to ensure clinical safety.

### 3.7. Peptide-Based Nanoparticles

Peptide-based nanoparticles (PNPs) have emerged as a promising strategy for melanoma therapy due to their biocompatibility, specificity, and ability to enhance drug delivery and immunomodulation. These nanoparticles consist of self-assembling peptides or peptide-polymer conjugates that form stable nanostructures into which therapeutic agents can be encapsulated, e.g., chemotherapeutics, targeted inhibitors, immunomodulators, and gene therapy [152]. The unique advantage of peptide-based nanoparticles lies in their high specificity for melanoma cells, which can be achieved by functionalizing them with tumor-specific peptides, such as RGD (arginine-glycine-aspartic acid), melittin and integrin targeting sequences that enhance selective accumulation in the tumor microenvironment [152]. This targeted approach significantly improves drug bioavailability, minimizes systemic toxicity, and improves therapeutic outcomes. In addition, peptide-based nanoparticles are often engineered with stimulatory properties that allow them to degrade in a controlled manner and release drugs when exposed to tumor-specific conditions, such as acidic pH, enzymatic activity, or redox imbalances [152].

In addition to drug delivery, peptide-based nanoparticles have shown significant potential for cancer immunotherapy by modulating the tumor’s immunological microenvironment. These nanoparticles can be engineered to deliver peptide-based vaccines, immune checkpoint inhibitors, or adjuvants that stimulate a robust anti-tumor immune response. For example, PNPs carrying tumor-associated antigen (TAA) peptides can enhance dendritic cell activation and promote T cell-mediated destruction of melanoma [153]. In addition, combination therapies combining peptide-based nanoparticles with immune checkpoint inhibitors (e.g., anti-PD-1/PD-L1 antibodies) have shown synergistic effects leading to increased tumor regression and prolonged survival in preclinical melanoma models [154]. Advances in nanotechnology and biotechnology have further optimized the design of these nanoparticles and improved their stability, penetration, and retention in melanoma tumors. Despite these advances, challenges such as scalability, stability, and regulatory approval remain, necessitating further research and clinical evaluation [155]. With further innovation, peptide-based nanoparticles have the potential to revolutionize melanoma treatment and provide safer, more effective, and personalized therapeutic strategies [156].

## 4. Challenges in Nanotechnology

The further development of nanotechnology-based drug delivery systems for melanoma therapy is associated with considerable challenges due to complex manufacturing processes, targeting accuracy, and physiological barriers (Table 2). The development of nanoparticles often involves complicated manufacturing processes that require specialized equipment, stringent environmental conditions, and skilled personnel. This complexity increases production costs and limits scalability and broad clinical application. In addition, nanoparticles face significant physiological obstacles, such as clearance through the RES, limited penetration through the dense tumor stroma, and suboptimal uptake by melanoma cells.

These obstacles require innovative strategies, such as surface modification with targeted ligands or using stimuli-responsive materials. However, given the heterogeneity of tumor biology and the variability of receptor expression, achieving precise targeting of melanoma cells while sparing healthy tissue remains challenging. Such limitations necessitate highly customized approaches that further increase development time and costs [183,184,185,186,187].

Stability, biocompatibility, and regulatory compliance are other critical challenges in this field. Nanoparticles must maintain physical and chemical stability during storage and systemic circulation to ensure therapeutic efficacy. However, many nanomaterials are prone to degradation or aggregation, which impairs their performance. In addition, ensuring biocompatibility to minimize immune reactions and toxicity is a constant concern, especially when using synthetic materials or complex formulations. Regulatory hurdles also slow down the translation of nanotechnology-based systems into clinical applications. Due to the novelty of these technologies, they must undergo rigorous safety, efficacy, and reproducibility testing, which extends approval timelines. In addition, scaling up nanoparticle production from laboratory conditions to industrial levels brings another set of challenges, including maintaining batch uniformity and consistent therapeutic performance. This scalability issue, combined with the high cost of development, limits access to these novel therapies, particularly in resource-constrained [180,181,182].

TME and patient-specific variability further complicate the application of nanotechnology in melanoma therapy. The hypoxic, acidic, and immunosuppressive conditions in the TME pose significant hurdles to effective drug delivery and therapeutic response. Dynamic and adaptable materials are needed to overcome these challenges, but their development is still in its infancy. In addition, genetic, immunological, and disease progression differences between patients require personalized nanoparticle systems, making the development and implementation of these therapies even more complex [19,183,184,185]. Long-term safety concerns also remain, particularly for non-biodegradable materials that can accumulate in vital organs and pose a risk of chronic toxicity [186,187,188]. Finally, ethical and social concerns, such as equitable access, environmental impacts of manufacturing, and potential misuse, underscore the far-reaching implications of advancing this technology. Addressing these multifaceted challenges requires an interdisciplinary approach that combines materials science, clinical research, and policymaking to ensure the successful integration of nanotechnology into melanoma treatment paradigms [189,190].

## 5. Future Perspectives

Nanotechnology has enormous potential to improve melanoma therapy by overcoming the limitations of conventional treatments and ushering in a new era of precision medicine [191,192,193,194]. The advent of diagnostic nanoparticles has fundamentally changed melanoma treatment by enabling early detection of metastatic tumors while delivering targeted drugs with fewer side effects [14,195,196]. These advances can significantly improve patient outcomes by enabling precise and controlled delivery of therapeutics. One of the most promising directions in nanotechnology-assisted melanoma therapy is the development of nanoparticles that take advantage of the properties of the tumor microenvironment, such as acidic pH or overexpression of certain receptors. These smart nanoparticles can ensure enhanced accumulation of the drug in the malignant tissue, thus improving therapeutic efficacy and reducing systemic toxicity [197]. In addition, the integration of stimulus-dependent nanocarriers, which release their payload in response to external stimuli, such as light, heat, or magnetic fields, offers the unique opportunity to achieve spatial and temporal control over drug delivery. This level of precision increases the safety and efficacy of treatments and can reduce the side effects associated with conventional chemotherapy [198].

Nanotechnology is expected to merge with advances in genetics, proteomics, and artificial intelligence (AI) in the coming years to further revolutionize melanoma therapy. AI-powered predictive models and genomic profiling may facilitate the personalization of nanoparticle-based therapies, allowing clinicians to customize treatment regimens based on an individual patient’s tumor profile [199,200,201]. This approach would represent a significant shift from a one-size-fits-all model to truly individualized cancer therapy. A major challenge in translating these innovations from research to clinical practice lies in the scalability of nanoparticle production and its integration into existing clinical workflows. Ensuring reproducibility, cost-effectiveness, and regulatory compliance will be critical to bridge the gap between laboratory research and practical application. In addition, the complexity of the tumor microenvironment poses a challenge to effective drug delivery, necessitating further research into multifunctional nanocarriers that can adapt to dynamic tumor conditions. Further development of multilevel targeting strategies, in which nanoparticles not only target melanoma cells but also modulate the surrounding tumor microenvironment, will be critical to overcoming drug resistance and improving treatment outcomes. In addition, the combination of nanotechnology with real-time imaging techniques has the potential to improve the accuracy of melanoma diagnosis and treatment monitoring.

Advances in materials science and nanotechnology have revolutionized melanoma therapy, offering innovative strategies to overcome the limitations of conventional treatments. The development of sophisticated drug delivery systems, including liposomes, dendrimers, and polymeric and metallic nanoparticles, has significantly improved drug solubility, stability, and targeted delivery. These nanocarriers utilize both passive and active targeting mechanisms, such as the enhanced permeability and retention (EPR) effect and ligand-based functionalization, to ensure precise drug delivery while minimizing systemic toxicity. The integration of stimuli-responsive nanoparticles and multi-step targeting strategies has further improved the precision of melanoma therapies, enabling controlled drug release in response to specific cues from the tumor microenvironment. These advances address critical challenges, such as drug resistance, poor tumor penetration, and treatment heterogeneity, and significantly improve therapeutic outcomes. In addition, the emergence of diagnostic platforms that combine treatment with real-time imaging holds the promise of personalized medicine, where therapies are tailored to the individual patient profile. Despite their transformative potential, challenges remain, including production scalability, biocompatibility, regulatory hurdles, and managing the complexity of the tumor microenvironment. However, ongoing research and interdisciplinary collaboration will continue to lead to breakthroughs in this area. Future developments in nanotechnology, combined with advances in bioengineering and precision oncology, promise safer, more effective, and more accessible treatments. These advances herald a new era in melanoma treatment, where cutting-edge nanotechnology innovations are redefining therapeutic approaches and improving patient outcomes.

## Figures and Tables

**Table 1:** Polymeric nanoparticles in melanoma therapy.

Drug Molecule	Description	References
Dacarbazine	Cholic acid (CA)-poly(lactide-co-glycolide)-b-polyethylene glycol (CA-PLGA-b-PEG) was used as a multifunctional polymeric carrier for the targeted delivery of dacarbazine (DTIC) to malignant melanoma (MM) cells. The system combined receptor-mediated uptake via the CA moiety, controlled release from the PLGA core, and improved biocompatibility through PEGylation. The DTIC-loaded NPs modified with the aptamer AS1411 (DTIC-NPs-Apt) showed improved targeting to the tumor, superior cytotoxicity, and minimal systemic toxicity, representing a promising therapeutic strategy for MM.	[122]
Dacarbazine + Quercetin	PLGA-based NPs were developed to co-administer dacarbazine (Dac) and quercetin (Qct) for melanoma therapy, considering the poor absorption, short half-life, and chemoresistance of Dac. These NPs improved skin permeation, lowered the IC50 of the drug, and inhibited melanoma cell migration, suggesting anti-metastatic potential. However, challenges such as low drug loading, stability of Dac, and different release profiles require further optimization for better efficacy.	[123]
Silibinin	Drug-induced hepatotoxicity limits the clinical use of many drugs. Silibinin (SBN) protects the liver but is poorly soluble in water. PLGA nanoparticles have been developed to sustainably release SBN and enhance antioxidant activity. In a melanoma mouse model with dacarbazine (DTIC), these NPs accumulated in the liver, reduced enzyme levels, prevented damage, and preserved function without compromising the efficacy of DTIC, providing a novel hepatoprotective strategy.	[124]
Paclitaxel	PLGA-based paclitaxel (PTX) NPs and films show promise for the treatment of melanoma. The NPs outperformed the films in terms of drug release, encapsulation, and efficacy. Both reduced tumor growth with minimal toxicity, with PTX release occurring by Fick’s diffusion, highlighting NPs as a safer option. Carboxymethyl chitosan NPs improved stability, bioavailability, and sustained release of PTX. They improved cellular uptake, increased cytotoxicity, and effectively inhibited tumor growth with less systemic toxicity, making them promising melanoma therapy.	[125,126]
Indocyanine green and Nexturastat A	PLGA-based nanoparticles (INAPs) encapsulated indocyanine green (ICG) for photothermal therapy and nexturastat A (NextA) for epigenetic therapy showed stable photothermal heating and effective histone deacetylases (HDAC) inhibition. They enhanced immune activation through the expression of costimulatory molecules and MHC class I in melanoma cells, which slowed tumor growth and improved survival in a melanoma model, highlighting their potential for synergistic cancer therapy.	[127]
Trametinib	Multifunctional PLGA NPs coated with T-cell membranes derived from 19LF6 hybridomas carrying melanoma-specific anti-gp100/HLA-A2 T cell receptor (TCR) were developed and loaded with the FDA-approved drug trametinib, a kinase inhibitor. These NPs exhibited high stability, compatibility, and sustained drug release, with cellular uptake increased in proportion to membrane/TCR content. They showed superior cancer-killing activity in vitro and a more than twofold increase in tumor retention in vivo compared to uncoated NPs.	[128]
Dacarbazine and Enoxaparin	Enoxaparin-coated dacarbazine-loaded chitosan nanoparticles (Enox-Dac-Chi NPs) showed high drug loading, sustained release, superior cytotoxicity, and enhanced antiangiogenic activity, highlighting their potential for the treatment of metastatic melanoma. Enox-Dac-Chi NPs showed superior cytotoxicity (IC50 of 59.60 µg/mL) against melanoma cells compared to dacarbazine alone or dacarbazine-loaded chitosan NPs and exhibited enhanced antiangiogenic activity, suggesting the role of enoxaparin in reducing melanoma metastasis.	[129]
Aminolevulinic acid derivatives	Chitosan nanoparticles improved the delivery of aminolevulinic acid (ALA) derivatives for the treatment of melanoma by combining photodynamic therapy (PDT) and electrochemotherapy (ECT). PDT resulted in localized tumor cell death, while ECT improved drug uptake, showing synergistic effects in reducing melanoma cell viability and tumor growth in vivo. These results make chitosan NPs a promising platform for dual melanoma treatment.	[130]
10-Hydroxycamptothecin	Positively charged chitosan-based 10-hydroxycamptothecin NPs (NPs/HCPT) were developed to improve tumor penetration and therapeutic efficacy of HCPT for the treatment of melanoma. The NPs/HCPT were characterized by dynamic light scattering and zeta potential analysis and showed effective cellular uptake, significant cytotoxicity against mouse melanoma cell lines (B16F10 and B16F1), and induced apoptosis, highlighting the potential of cationic NPs/HCPT as a promising nano system for melanoma chemotherapy.	[131]
Genistein	Poly-caprolactone (PCL) NPs (PN) stabilized with either D-α-tocopherol polyethylene glycol 1000 succinate (TPGS) or poloxamer 407 were developed to encapsulate genistein (GN) for melanoma therapy. PNTPGS-GN showed higher cellular uptake (80%) and higher encapsulation efficiency (77.95%), while PNPol-GN exhibited lower toxicity but lower uptake. Thermal and spectroscopic analyses confirm stability and compatibility. These results suggest that PCL NPs are a promising nanomedicine for the treatment of melanoma that warrants further preclinical studies.	[132]
Curcumin	This study investigated the effects of orally administered chitosan-coated polycaprolactone NPs containing curcumin on metastatic melanoma in the lung. The NPs effectively reduced the viability of B16F10 melanoma cells, triggered apoptosis, and regulated the expression of metalloproteinases, which are crucial for the migration and proliferation of cancer cells. These results highlight the potential of chitosan-coated NPs loaded with curcumin as a promising therapeutic strategy for metastatic melanoma.	[133]
Metformin	Chitosan polymeric NPs gel loaded with metformin was investigated for the topical treatment of melanoma. The formulation showed sustained drug release, excellent biocompatibility, and effective penetration into melanoma cells in vitro. Ex vivo studies confirmed that metformin better penetrates and remains in the skin, optimizing its local delivery. The gel significantly reduced the viability of melanoma cells and exhibited promising anti-cancer activity, indicating its potential as an effective topical therapy for melanoma.	[134]

**Table 2:** Challenges in nanotechnology for precision melanoma therapy.

S.no	Challenge	Description	References
1	Complex Fabrication Processes	Intricate techniques that require specialized equipment, strict conditions, and skilled personnel, resulting in increased production costs and limited scalability.	[157,158,159]
2	Physiological Barriers	In the treatment of melanoma, NPs face physiological challenges, including rapid clearance by the reticuloendothelial system (RES), low tumor accumulation, and limited penetration through the dense stroma. Low cellular uptake further reduces efficacy, often requiring advanced surface modifications for improved targeting and delivery.	[160,161,162]
3	Stability and Biocompatibility	The stability of nanoparticles is crucial for effective melanoma therapy to prevent aggregation, degradation, and loss of function during storage and circulation. They must resist enzymatic degradation while ensuring efficient drug delivery. Stability and biocompatibility help to cope with the acidic pH, high pressure, and oxidative stress of melanoma and improve precision and safety. Strategic material selection and surface modifications are critical for reducing immune responses and toxicity to optimize nanoparticle-based treatments.	[163,164]
4	Targeting Precision	The heterogeneity of tumors and the differential expression of receptors pose a challenge for nanoparticle-based melanoma therapies and require personalized targeting strategies to ensure precise drug delivery while minimizing the impact on healthy tissue. The incorporation of targeting ligands, antibodies, or peptides increases specificity but also makes development and manufacturing more complex and requires extensive research, time, and resources to optimize therapeutic efficacy.	[165,166,167,168,169]
5	Drug Loading and Release	Optimizing drug loading and sustained release in melanoma therapy based on nanoparticles improves efficacy by reducing dosage and side effects while maintaining uniform drug levels at the tumor site. Careful material selection and formulation prevent premature release or poor encapsulation, ensuring better treatment outcomes and patient compliance.	[169,170]
6	Tumor Microenvironment (TME) Resistance	TME presents significant resistance to effective nanoparticle delivery and therapy. TME in melanoma poses a challenge for nanoparticle treatment due to hypoxia, acidic pH, and immunosuppression, which compromises treatment efficacy. Adaptive nanoparticles, such as pH-sensitive or oxygen-releasing systems, are critical to improving drug stability, immune response, and targeted delivery for better therapeutic outcomes.	[171,172,173]
7	Short Circulation Half-Life	The short half-life of nanoparticles in the bloodstream significantly limits their therapeutic efficacy, as rapid elimination by the liver, spleen, or kidneys reduces their ability to accumulate at tumor sites. This premature elimination prevents sufficient interaction with melanoma cells and thus impairs drug delivery and efficacy. The short half-life of nanoparticles limits melanoma therapy as they are rapidly degraded, which reduces accumulation in the tumor and the efficacy of the drug. Surface modifications with PEG or biocompatible coatings help prevent them from being recognized by the immune system, prolong circulation time, and improve stability for effective drug delivery.	[174,175,176,177]
8	Long-Term Safety Concerns	The long-term safety of nanoparticle-based drugs remains a challenge, as slowly degradable or non-biodegradable materials can accumulate in vital organs and cause chronic toxicity. Understanding biodegradability, degradation mechanisms, and toxicological effects is critical to ensuring clinical safety and minimizing risks from prolonged exposure.	[178,179]
9	Multidrug Resistance (MDR)	MDR in melanoma therapy is a critical challenge as it significantly limits the efficacy of both conventional drugs and nanoparticle-based delivery systems. Mechanisms such as overexpression of efflux pumps, enhanced DNA repair, and evasion of apoptosis allow melanoma cells to survive and proliferate despite treatment. Advanced approaches are needed to combat MDR, such as co-delivery systems simultaneously releasing multiple drugs targeting different signaling pathways or novel therapeutic combinations inhibiting resistance mechanisms. These strategies improve treatment outcomes and require innovative nanoparticle designs to combat melanoma-specific resistance.	[180,181,182]
